# AAV-Mediated Gene Therapy for Choroideremia: Preclinical Studies in Personalized Models

**DOI:** 10.1371/journal.pone.0061396

**Published:** 2013-05-07

**Authors:** Vidyullatha Vasireddy, Jason A. Mills, Rajashekhar Gaddameedi, Etiena Basner-Tschakarjan, Monika Kohnke, Aaron D. Black, Krill Alexandrov, Shangzhen Zhou, Albert M. Maguire, Daniel C. Chung, Helen Mac, Lisa Sullivan, Paul Gadue, Jeannette L. Bennicelli, Deborah L. French, Jean Bennett

**Affiliations:** 1 F.M. Kirby Center for Molecular Ophthalmology, Scheie Eye Institute, University of Pennsylvania School of Medicine, Philadelphia, Pennsylvania, United States of America; 2 Center for Cellular and Molecular Therapeutics, The Children’s Hospital of Philadelphia, Philadelphia, Pennsylvania, United States of America; 3 Institute for Molecular Bioscience, University of Queensland, St. Lucia, Queensland, Australia; 4 Department of Anatomic Pathology, The Children’s Hospital of Philadelphia, Philadelphia, Pennsylvania, United States of America; 5 Department of Pathology and Laboratory Medicine, The Children’s Hospital of Philadelphia, Philadelphia, Pennsylvania, United States of America; University of Florida, United States of America

## Abstract

Choroideremia (CHM) is an X- linked retinal degeneration that is symptomatic in the 1^st^ or 2^nd^ decade of life causing nyctalopia and loss of peripheral vision. The disease progresses through mid-life, when most patients become blind. CHM is a favorable target for gene augmentation therapy, as the disease is due to loss of function of a protein necessary for retinal cell health, Rab Escort Protein 1 (REP1).The *CHM* cDNA can be packaged in recombinant adeno-associated virus (rAAV), which has an established track record in human gene therapy studies, and, in addition, there are sensitive and quantitative assays to document REP1 activity. An animal model that accurately reflects the human condition is not available. In this study, we tested the ability to restore REP1 function in personalized *in vitro* models of CHM: lymphoblasts and induced pluripotent stems cells (iPSCs) from human patients. The initial step of evaluating safety of the treatment was carried out by evaluating for acute retinal histopathologic effects in normal-sighted mice and no obvious toxicity was identified. Delivery of the *CHM* cDNA to affected cells restores REP1 enzymatic activity and also restores proper protein trafficking. The gene transfer is efficient and the preliminary safety data are encouraging. These studies pave the way for a human clinical trial of gene therapy for CHM.

## Introduction

Choroideremia (CHM) is an X- linked inherited retinal disease characterized by the degeneration of photoreceptors, retinal pigment epithelium (RPE) and choriocapillaris. Symptoms develop in the 1^st^ or 2^nd^ decade of life with complaints of poor night vision (nyctalopia) and progressive loss of peripheral vision. Visual fields constrict as the disease progresses. This culminates with loss of central vision (visual acuity) and blindness as early as the fourth decade of life [1], [2], [3], [4].

The choroideremia gene, *CHM*, encodes Rab Escort Protein-1 (REP-1), a 653 amino acid protein thought to be involved in membrane trafficking [5], [6]. Since the *CHM* locus is on the X-chromosome, choroideremia is typically only diagnosed in males. Although female carriers of the disease are usually asymptomatic, retinal exams often reveal a patchy degeneration of the retina and RPE and female individuals can be affected depending on the extent of X-inactivation of the normal X chromosome (lyonization) [7], [8].

REP-1 plays a key role in the post-translational lipid modification of Rab small GTPases (RABs), members of the Ras superfamily which, when integrated with membranes, serve as controllers of tethering, docking, and fusion. In humans, more than 60 RAB proteins have been identified to date [9]. Before newly produced RAB proteins can integrate into membranes, they must be post-translationally modified through the addition of prenyl groups to 1–2 cysteines located near their C-termini [10], [11]. In order for prenylation to occur, REP-1 must associate with Rab GTPases and present them to Rab geranylgeranyltransferase (Rab GGTase). Prenylated RAB proteins are escorted by REP-1 to their target membrane [12] where REP-1 then disassociates and returns to the cytosol. Lack of REP-1 as observed in CHM, is thought to lead to the accumulation of unprenylated RAB proteins and the eventual death of the affected cells [13], [14], [15]. In humans and other mammals, an autosomal *CHM*-like gene encodes a protein product, *REP-2* that functions very similarly to *REP-1*, and is thought to substitute for *REP-1* in most of the tissues of CHM patients, but not in the retina. Moreover REP-2 does not assist in the prenylation of RABs to the same extent as REP-1. In fact, some proteins, such as RAB27, are specifically and solely prenylated by REP-1 [16], [17], [18].

Because the disease is slowly progressive and does not affect longevity, and because CHM can be identified by a unique fundus appearance in both patients and carriers, many retina specialists have diagnosed this disease despite its rarity (estimated prevalence of 1∶50,000–1∶100,000 people (http://ghr.nlm.nih.gov/condition/choroideremia). Many features of choroideremia and its underlying biology make this disease an ideal candidate for retinal gene augmentation therapy. First, the disease phenotype results from loss of function. So far there are 113 known mutations in *CHM*, including nonsense mutations, splicing mutations, deletions, and insertions (http://www.retina-international.org/sci-news/databases/mutation-database/chm-mutation [19], [20], [21], and all are predicted to result in loss-of-function of REP-1. Second, there are no constraints to packaging of *CHM* in recombinant adeno-associated virus (rAAV) since the size of a *CHM* expression cassette falls within the 4.7 kb packaging limit for rAAV. Third, only retinal tissues are affected in individuals with *CHM* mutations, allowing use of limited amounts of vector to a very small anatomic target despite the fact that this gene is normally expressed throughout the body. Although the RPE is thought to be the primary cell type affected in the disease, additional retinal cell types express the *CHM* gene [22] and could also be therapeutic targets. Finally, there is now a large body of safety data relating to gene transfer of wild type cDNAs, particularly with respect to the retina [23]. There are currently 13 different human retinal gene therapy clinical trials in progress, including a recently initiated Phase III (FDA drug approval) trial (clinicaltrials.gov). The majority of these studies utilize AAV2 as the gene transfer vector. Thus, there is now abundant data regarding the safety of subretinal administration of AAV2, including the safety of readministration to the contralateral eye [24], [25].

For the reasons outlined above, we believe that choroideremia is an excellent target for gene augmentation therapy. Indeed, one choroideremia clinical trial has already commenced (http://www.blindness.org/index.php?view=article&id=2950%3Afirst-patient-treated-in-choroideremia-gene-therapy-clinical-trial-in-uk_content&Itemid=124). The studies described here evaluate the efficacy and *in vitro* safety profile of a vector that we consider as an additional candidate for a clinical trial. One of the challenges in developing gene therapy for CHM is that the engineered animal model does not closely resemble the functional and morphologic manifestations of the disease with complete accuracy. Further, this model is not readily available. Therefore, we have developed proof-of-concept using *in vitro* models, including induced pluripotent stem cells (iPSCs) derived from individuals with CHM. Preliminary safety studies have also been carried out in normal-sighted mice. The results demonstrate robust reversal of the biochemical and protein trafficking deficits in the cell models with an encouraging safety profile. Besides demonstrating proof-of-concept of gene augmentation therapy for choroideremia, this study could serve as a model in future applications as to how to carry out proof-of-concept studies when a relevant animal model is lacking.

## Materials and Methods

All research involving human participants was approved by the University of Pennsylvania and The Children’s Hospital of Philadelphia institutional review boards. Written informed consent was obtained from all human participants after discussions of the procedures and alternatives as well as potential risks and benefits. The consent process was witnessed by an adult caregiver. All clinical investigations were conducted according to the principles expressed in the declaration of Helsinki. No minors/children were included as participants. All animal work was conducted according to relevant national and international guidelines and steps were taken to be sure there was minimal suffering. All animal studies were approved by the University of Pennsylvania Institutional Animal Care and Use Committee: IACUC #200902.

### 1.1. Generation of a Recombinant Adeno-associated Virus (AAV) Carrying the Full-length Human *REP-1*-encoding cDNA

Recombinant AAV was generated by the Center for Cellular and Molecular Therapeutics at The Children’s Hospital of Philadelphia (CHOP) after triple transfection of HEK293T cells with pAAV2.CBAe.hCHM and helper plasmids and was isolated and purified as previously described [26], [27]. The purified virus, named AAV2.hCHM, was stored frozen (−80°C) in sterile tubes until use. http://www.sciencedirect.com/science/article/pii/S0042698902003899 - gr1.

### 1.2. Non-iPSC Cell Lines and Tissue Culture Conditions

CHO cells were cultured in DMEM/F12 medium with 10% FBS and 1% penicillin/streptomycin (Invitrogen, Carlsbad,CA). Fibroblasts were grown as primary cultures in 10% FBS containing DMEM with 1% penicillin/streptomycin and 2% glutamine (Invitrogen, Carlsbad, CA). All cells were grown at 37°C and 5% CO_2_. Transfections were carried out using Lipofectamine 2000 (Invitrogen, Carlsbad, CA) or Fugene-6 transfection reagent (Roche applied Sciences, Indiana Police, IN) according to the manufacturer’s protocol. The mutation in fibroblasts from CPF1 had been previously identified [28].

### 1.3. Immunofluorescence Analysis

Immunofluorescence was performed as previously described [29] using monoclonal REP-1 antibody (2F1, 1∶500, Santa Cruz Biotechnology, Santa Cruz, CA). Anti-mouse Alexafluor 488 (1∶1000) or 594(1∶3000), anti-rabbit Alexafluor 488 (1∶1000) or 594 (1∶3000) labeled antibodies are used as secondary antibodies (Invitrogen, Carlsbad, CA). Fluorescence was visualized with a Zeiss Axio Imager-M2 microscope and captured using an Axiocam MRm camera with Axiovision software and a Zeiss confocal microscope (Carl Zeiss Microscopy, LLC, Thornwood, NY).

### 1.4. Vector dose Response in CHO-K1 Cells

Aliquots of CHO cells were infected with various concentrations of AAV2.hCHM ranging from 1×10^3^ to 2×10^5^ vg/cell. Cells were harvested 48 h post-transduction and processed for Western blot and immunofluorescence analysis [29], [30].

### 1.5. Immunoblotting

Western blot analysis was performed using anti-REP-1 2F1 antibody (2F1, 1∶1000 dilution) as primary antibody and a secondary HRP conjugated anti-mouse IgG antibody (Amersham Biosciences, Piscataway, NJ) at a concentration of 1∶5000 [30]. The blots were developed by chemiluminescence using ECL reagents according to the manufacturer’s instructions (Amersham Biosciences, Piscataway, NJ).

### 1.6. Infection of Cultured Fibroblasts

Primary skin fibroblast cells (CPF1 cells) provided by a CHM patient were infected with AAV2.hCHM at an MOI of 2×10^5^ and processed for immunofluorescence or immunoblotting as described above. Isolation and characterization of CPF1 cells, provided after the subject had given written consent, was described earlier [27].

### 1.7. Detection of Apoptotic Nuclei in Transduced Cells

Apoptosis was assessed using the *In Situ* Cell Death Detection Kit with Tetramethylrhodamine red labeling (TMR red) (Roche Applied Sciences, Indianapolis, IN) and a 4′,6-diamidino-2-phenylindole (DAPI) counterstain. TUNEL staining was performed on AAV2. hCHMtransduced cells grown on microscope chamber slides (Labteck, Scotts Valley, CA) according to the manufacturer’s protocol (Roche applied Sciences, Indianapolis, IN). Positive controls were generated by incubating the cells with DNase I to induce strand breakage, while negative controls lacked the Tdt enzyme necessary for TMR red labeling.

### 1.8. Generation of Human iPSCs

Human peripheral blood mononuclear cells (PBMCs) were isolated from whole blood by centrifugation on a FICOLL gradient or in cell preparation tubes (CPT tubes) cultured in expansion media consisting of QBSF-60 (Invitrogen, Carlsbad, CA) media supplemented with cytokines and hormone as previously described [31], [32]. Human studies were approved by the University of Pennsylvania Institutional Review Board (IRB; # 808828) and also the CHOP IRB board (#09-007042). The media was replenished every 2–3 days for a period of 7–9 days until the cells entered a stage of exponential growth. For reprogramming, expanded PBMCs were transduced with the rTTA lentivirus and doxycycline inducible “stem cell cassette” in the lentivirus vector delivering *OCT4, KLF4, SOX2*, and *cMyc* cDNA and microRNA 302/367 cluster driven by the TetO/CMV promoter. Cells were grown in expansion media supplemented with polybrene (5 ug/ml; Sigma-Aldrich, St.Louis, MO) [33]. The cells were incubated for 20–24 h at 37°C. Infected cells were then washed, and placed in expansion media supplemented with 1 ug/ml of doxycycline (DOX). After 48 h, cells were resuspended in Iscove’s modified Dolbecco’s medium (IMDM) with 10% fetal bovine serum(FBS), penicillin/streptomycin, L-glutamine, beta-mercaptoethanol, nonessential amino acids, 4 ng/ml of basic fibroblast growth factor (bFGF), and 1 ug/ml of DOX (Sigma-Aldrich, St.Louis, MO) then moved onto matrigel-coated (BD Biosciences, San Jose, CA) mouse embryonic fibroblast plates (MEF plates). Cells remained in this media for 10 days, then were transferred to human embryonic stem cell (hESC) media (DMEM/F12, 20% knockout serum replacement, nonessential amino acid, 4 ng/ml bFGF, 0.001% beta-mercaptoethanol, penicillin/streptomycin, L-glutamine, and 1 ug/ml of DOX (Invitrogen, Carlsbad, CA). After 4 weeks, iPSC-like colonies were manually picked and expanded on matrigel-coated MEF plates for 6 passages, then transitioned to 0.1% gelatin-coated MEF plates for a minimum of 15 passages. Characterization of iPSCs was based on surface antigen expression using flow cytometry as previously reported [31].

Characterization of iPSCs was based on surface antigen expression measured by flow cytometry using antibodies against SSEA3+, SSEA4+, TRA-1-60, and TRA-1-81 (Biolegend, San Diego, CA), and RT-qPCR analysis included pluripotency expression markers: *DMNT3B, ABCG2, REX1, OCT4, SOX2, NANOG, cMYC, KLF4*. Karyotyping of iPSCs was carried out by G banding to produce a visible karyotype. For teratoma formation, a minimum of 1×10^6^ cells was resuspended in 200 ul of (1∶1) matrigel and subsequently injected into the neck of Fox Chase SCID Beige Mice. Mice were sacrificed 6–9 weeks later, and teratomas were isolated and processed for histological analysis.

### 1.9. Flow Cytometric Evaluation of the Expression of REP-1 in CPS1 Cells

The iPSCs generated from CHM individuals (CPS1) were transduced with AAV2. hCHM at a multiplicity of infection (MOI) of 2×10^5^. After 48 h, both control and transduced cells were dissociated and the cells were fixed, permeabilized and stained in 1X saponin buffer as published [31]. Staining was performed with anti-human REP-1 2F1 antibody(Santa Cruz Biotechnology, Santa Cruz, CA, 1∶200 dilution) followed by goat anti-mouse IgG1-DyLight649 (Jackson ImmunoResearch, West Grove, PA, 1∶400 dilution). Cells were counted on a FACS Cantos II flow cytometer (BD Biosciences, San Jose, CA) and the results analyzed using FlowJo software (Tree Star Inc., Ashland, OR).

### 2.0. Intracellular Imaging

Control and iPSCs infected with AAV2. hCHM at an MOI of 2×10^5^ were collected and fixed with 2% paraformaldehyde and permeabilized with permeabilization/wash buffer (P/W) (BD Biosciences, San Jose, CA) according to manufacturer’s instructions. Intracellular staining for the RAB27 and REP-1 proteins was performed using the mouse 2F1 primary antibody at a dilution of 1∶100 and rabbit anti-RAB27 primary antibody (Sigma-Aldrich, Saint Louis, MO) also at a dilution of 1∶100. After washing, fluorophore-conjugated secondary antibodies were added at 1∶1500 anti-mouse-Alexa 647 and 1∶500 anti-rabbit-PE. Cells were then washed, fixed and images were acquired with an AmnisX ImageStream instrument (Amnis Corporation, Seattle, WA). Imaging of protein expression and intracellular localization was performed at a 40× magnification. At least 10,000 cells were acquired for each condition. Data analyses included protein expression and functionality of REP-1. The functionality of REP-1 was monitored by intracellular RAB27 localization. A surface mask was defined with the brightfield image using Amnis software (www.amnis.com) and the percent of RAB27 localized within the mask was measured. The cells were analyzed using IDEAS software (Amnis Corporation, Seattle, WA). As the image stream collects multiple fluorescence images per cell, localization of cellular markers to specific subcellular compartments can be achieved in a quantitative manner. The ImageStream imaging flow cytometer acquires 6 different channels of images which include bright field, dark field and 4 channels of fluorescence images. The pictures captured in each of the channels are in a separate spatial registry, which enables the measurement of fluorescent signal in sub cellular compartments. Correlation analysis measures the similarity of the images between channels.

### 2.1. *In vitro* Prenylation Assay

An *in vitro* prenylation assay was performed using [^3^H]-geranylgeranyl pyrophosphate (GGPP) (Perkin Elmer, Boston, MA, USA) as a prenyl group donor, in the presence of recombinant Rab geranylgeranyl transferease and RAB27 (custom order from Blue Sky Biotech, Worcester, MA) as described [30]. Incorporation of radiolabeled prenyl groups into the RAB27 protein was measured by scintillation counting. For consistency the control values were normalized to 100 and used as the base value. All experiments were performed in triplicate, and statistical comparison of prenylation between experimental and control groups was evaluated using the two-tailed unpaired student’s *t*-test.

### 2.2. Intraocular Administration of AAV2. hCHM

Subretinal injections of AAV2. hCHM were performed in a cohort of 8 week old C57Bl/6 mice at a dose of 2.7×10^10^ vg. Animal studies were carried out in strict accordance with the recommendations in the Guide for the Care and Use of Laboratory Animals of the National Institutes of Health. The protocol was approved by the University of Pennsylvania institutional animal care and use committee (IACUC #200902). All surgery was performed under ketamine/Xylazine anesthesia, and all efforts were made to minimize suffering. Contralateral eyes were used as uninjected controls. Injections were monitored by direct visualization through the operating microscope. Evaluation of the expression of REP-1 was performed 3 weeks after injection.

### 2.3. Retina Fixation and Cryosectioning

Mice injected with AAV2. hCHM were sacrificed after 3 weeks when eyes were harvested and fixed in 4% PFA. Eyes were then embedded in Optimal Cutting Temperature media (Fisher Scientific), and frozen. Cryosections were then made using a Leica CM1850 cryostat (Leica Microsystems, Wetzlar, Germany) and nuclear layers were stained with DAPI.

### 2.4. Statistics

Data are presented as the mean ± Standard deviation (SD). *P* values were calculated using the 2-tailed Student’s *t* test. A P-value less than 0.05 is considered statistically significant.

## Results

### Generation and Preliminary Characterization of the Proviral Plasmid, pAAV2.CBAe.hCHM

#### Generation of pAAV2.CBAe.hCHM

**Figure 1:**
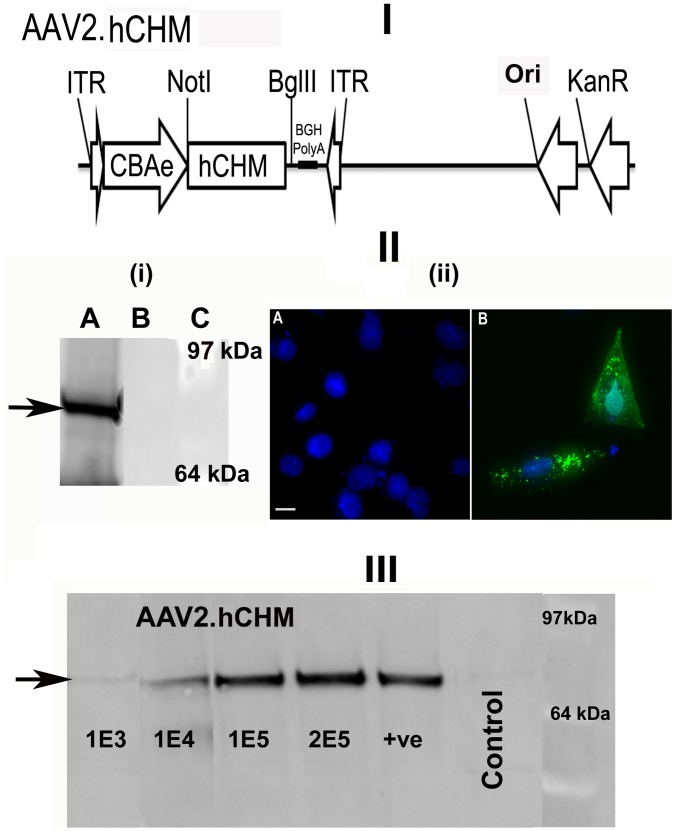
Generation and Characterization of AAV2. hCHM. I). Schematic of the AAV proviral plasmid carrying human *CHM*. under the control of the cytomegalovirus enhancer chicken beta actin (eCBA) promoter. ITR: Inverted terminal repeats; Ori: Replication origin; KanR: Kanamycin resistance gene. II) i) Immunoblot and ii) fluorescent analysis reveals REP-1 protein in CHO cells transfected with pAAV2.hCHM. Lane A: Transfected cell (25 ug protein), B: Control (untransfected) cells, C- protein marker. Immunocytochemical analysis revealed the localization of REP-1 to the cytosolic region (II-ii-B; Green). No REP1 is observed in control cells (II-ii-A). Nuclei are stained with DAPI and appear blue. Scale bar is 50 uM. **III)** Immunoblot analysis of CHO cells infected with 1E3-2E5 viral genomes (vg) of AAV2. hCHM show an increase in REP-1 protein (indicated by arrow) proportional to the titer. Positive (+ve) control: pAAV2. hCHM-transfected CHO cell lysate.

Proviral plasmid pAAV2.CBAe.hCHM was generated and features wild type human *CHM* cDNA (*hCHM*) under the control of a hybrid, cytomegalovirus enhancer-chicken beta actin promoter (CBAe) (figure 1–I). The plasmid includes a kanamycin resistance gene and a 5.1 kb stuffer sequence to prevent reverse packaging of non-transgene containing sequence from the plasmid backbone. Sequence analyses confirmed the absence of potential mutations introduced during cloning. This plasmid will be available to investigators for the purpose of academic, non-commercial research in adherence to PLOS ONE policies, but also in a manner that is consistent with and subjugate to the most current US FDA regulations.

#### Expression mediated by pAAV2.CBAe.hCHM in cultured cells

For initial tests of the proviral plasmid, transient transfections were carried out in Chinese hamster ovary (CHO) cells. Western blot analysis of total cell extracts using a monoclonal human REP-1-specific antibody, revealed expression of hREP-1 protein in transfected cells but not in untransfected control cells (figure 1-II-i, arrow). Immunofluorescence analysis of cells grown in monolayers revealed strong hREP-1 protein expression in the cytoplasm (figure 1-II-iiB) of transfected, but not control cells (figure 1-II-iiA). Recombinant AAV2. hCHM (virus) -mediated delivery of the CHM gene resulted in a dose-dependent response in REP-1 protein expression when the multiplicity of infection (MOI) ranged from 1×10^3^ to 2×10^5^ (arrow in figure 1-III).

### Generation of *in vitro* Models of Choroideremia and Restoration of REP-1 Function using AAV2. hCHM

#### Generation and characterization of cell lines derived from individuals with CHM

**Figure 2:**
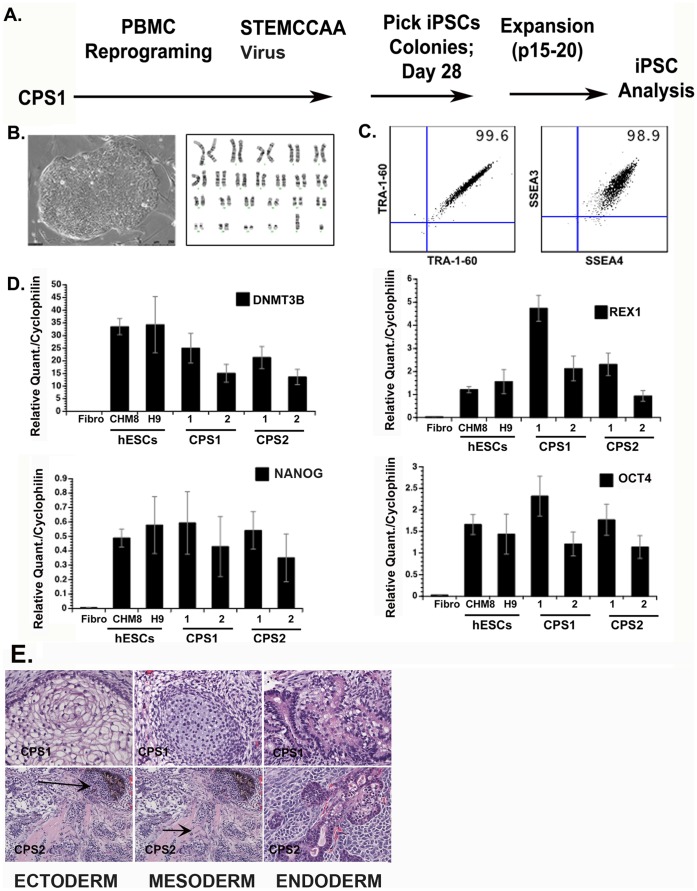
Characterization of choroideremia (CHM) iPSCs (CPS1 cells). Plots representative of 4 peripheral blood (PBMC)-derived iPSC clones. (A) iPSC reprogramming timeline (B) phase contrast image of established iPS clone with normal G-banded karyotype (C) Extracellular pluripotency markers; SSEA3/SSEA4, TRA-1-60*;* (D) Molecular profile of iPSC lines. Real-time PCR analysis shows expression of common pluripotency genes: *DNMT3B, REX1, OCT4, NANOG*. (E) Hematoxylin and eosin staining of CHM iPSC-derived teratomas displays 3 germ layers; endoderm, mesoderm, ectoderm. Arrows in panel E point towards two different germ layers in one image.

Two unrelated individuals with clinical diagnoses of choroideremia consented to submit blood samples for the generation of blood-derived cell lines and for molecular diagnoses. These cell lines are available except where this would breach confidentiality rules related to human subject research. For the generation of induced pluripotent stem cell lines (iPSc), peripheral blood mononuclear cells were transduced using the STEMCCA lentiviral vector containing the four Yamanaka reprogramming genes [31]. Cell lines from the two individuals were designated as CPS1 and CPS2, respectively. A schematic for the generation of CPS1 iPSCs is shown in figure 2A. A normal G-banded karyotype is shown in figure 2B. The iPSC lines were characterized for standard quality control criteria such as morphology (figure 2B) surface expressed pluripotency markers such as SSEA-4, and TRA-1-60 (figure 2C) [34], and gene expression of pluripotency markers such as *DNMT3B, REX-1, OCT4*, and *NANOG* (figure 2D) [35], [36]. All clones were maintained in culture for a minimum of sixteen passages before analyses to erase residual epigenetic memory associated with the cell of origin [32], [37]. The pluripotency ability of CPS clones was assessed by teratoma formation assay, generating each germ layer (ectoderm, mesoderm, and endoderm) (figure 2E).

Molecular genetic testing revealed a premature stop codon (Arg555 → stop) mutation in DNA from CPS1 was retained leading to a carboxy-terminal truncation of 99 amino acids [28], [30] mutation that is predicted to destabilize the REP-1 protein. CPS2 has an L550P missense mutation. Previous work using *in silico* analysis of the L550P mutation suggests that the proline residue at position 550 destabilizes the beta-structural elements, and REP-1 tertiary structure [14]. The loss of REP-1 protein was confirmed by western blot analysis in both cell lines.

**Figure 3:**
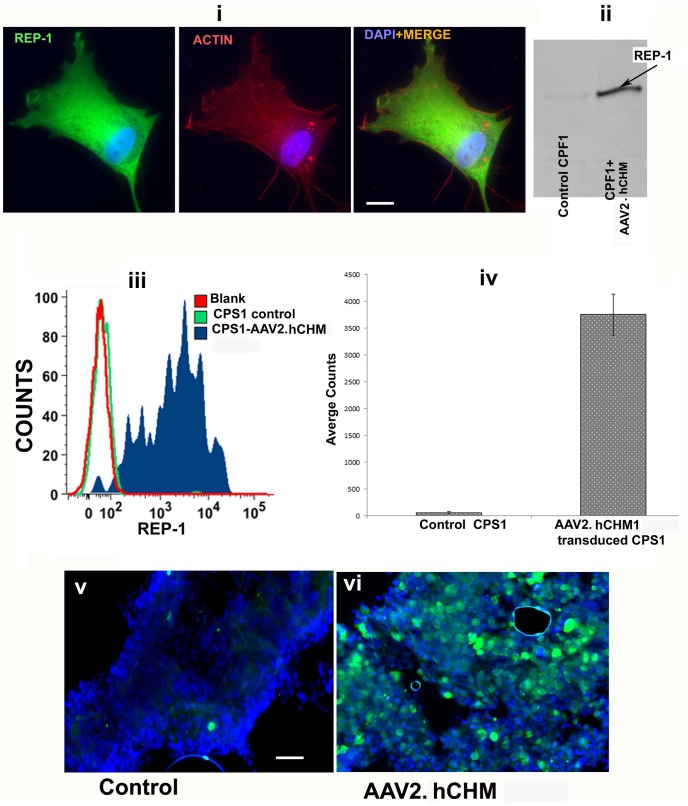
REP-1 protein is produced by fibroblasts [CPF1 (i, ii)] and iPSCs [CPF2 (iii, iv)] following AAV2. **hCHM infection**: Fibroblasts and iPSCs of CHM individuals were infected with 2×10^5^ vg/cell AAV2. hCHM and production of REP-1 was assessed by immunofluorescence (i); western blot analysis (ii); and flow cytometry (iii, iv). Cytoplasmic distribution pattern of REP-1 was confirmed by co-staining with anti-actin antibody (i). Western blot analysis (ii) further confirmed the presence of REP-1 protein. FACS analysis of CHM iPSCs infected with AAV2. hCHM showed a high level of REP-1 protein compared to untransduced controls (iii,iv). Immunofluorescence showed high levels of REP1 protein in cells infected with AAV2. hCHM (vi) compared with controls (v). Nuclei are stained with DAPI. Scale bar is 50 uM. Data are representative of 3 independent experiments.

A line of fibroblasts corresponding to the iPSC line CPS1, named CPF1, was also generated as previously described. [30] *Restoration of function mediated by AAV2. hCHM in vitro in CHM patient-derived cells*: Patient-derived fibroblasts were infected with AAV2. hCHM at an MOI of 2×10^5^ vg/cell and analyzed for the expression of REP-1 protein by immunofluorescence and western blot analysis. Representative results are shown for CPF1 (figure 3). Immunofluorescence analysis showed a lack of expression of REP-1 prior to exposure to AAV2. hCHM (data not shown). After infection, there was a predominant cytosolic localization of exogenous REP-1 (figure 3-i), as demonstrated with co-staining for actin. Immunoblot analysis of cells before and after infection with AAV2. hCHM demonstrated a lack of REP-1 in untreated cells whereas abundant REP-1 protein was present in treated cells (figure 3-ii, arrow).

Expression of REP-1 was also demonstrated in both iPS cell lines by flow cytometry, and representative results are shown for CPS1. While REP-1 was present at minimal levels in uninfected CPS1 cells, there was an approximately 50-fold increase in expression in AAV2. hCHM-treated cells (figure 3–iii, iv). As expected, immunofluorescence analysis of cultured, adherent cells showed high levels of REP-1 in CPS1 cells infected with an MOI of 2×10^5^ vg/cell AAV2.hCHM (figure 3–v, vi). Further comparison of the level of expression of REP-1 after infection of iPSCs was 35–40% greater than that obtained after infection of fibroblasts (figure A in file S1). The higher transduction efficiency of iPSCs with AAV2 compared to fibroblasts was further confirmed by infecting both wild type fibroblasts and iPSCs with AAV2-GFP. Immunofluorescence analysis for GFP revealed a 36% increase in the GFP expression in iPS cells compared to fibroblasts (figure A in file S1). In summary, these results not only confirm the high expression of REP-1 in AAV2-infected CHM iPSCs but also reveal a significant improvement in the transduction efficiency mediated by AAV2 in iPSCs compared to fibroblasts. Due to the improved expression and feasibility, iPS cells are an excellent model with which to establish the proof-of-principle for gene based therapy for choroideremia.

**Figure 4:**
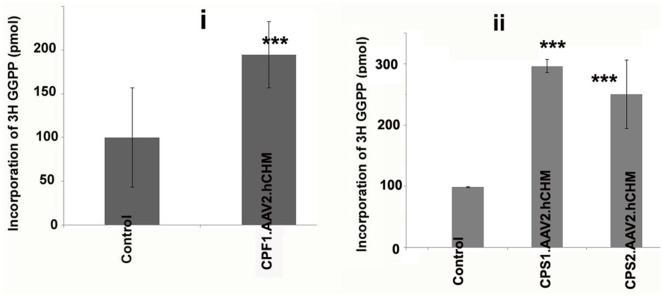
Prenylation activity in fibroblasts (CPF1) (i) and iPSCs (CPS1, CPS2) (ii) cultured from CHM individuals is restored following infection with AAV2. **hCHM.** Prenylation assay was performed using the cytosolic fraction of cells transduced with AAV2. hCHM and from untreated affected cells (Control). Cell lysates were incubated with RabGGTase, RAB27 and [3H]-labeled GGPP. A significant increase (P<0.02) in the prenylation activity of exogenous REP-1 was observed in both CPF1 (2 fold) and CPS1, CPS2 cells (∼3 fold).

To determine whether infection with AAV2. hCHM restores REP-1 function in cells of patients with loss of function *CHM* mutations, CPF1, CPS1, and CPS2 cells were infected with AAV2. hCHM at an MOI of 2×10^5^. Forty-eight hours post infection, cells were harvested and cytosolic fractions of the cells were isolated and used to perform *in vitro* prenylation assays in which the cytosolic cellular fraction served as the REP-1 protein source, RabGGTase, [^3^H]-GGPP as the prenyl group donor, and recombinant RAB27 as the substrate, as described previously [30], [38], [39]. We observed a significant increase in the prenylation of RAB27a in the fibroblast line, CPF1 (∼2 fold, 3-i; p = 0.01) and the iPSC lines, CPS1 and CPS2 (∼3 fold, figure 3-ii; p = 0.0015) cells transduced with AAV2. hCHM compared to the controls (figure 4-i,ii).

### Expression of Exogenous REP-1 Improves the Trafficking of RAB27

**Figure 5:**
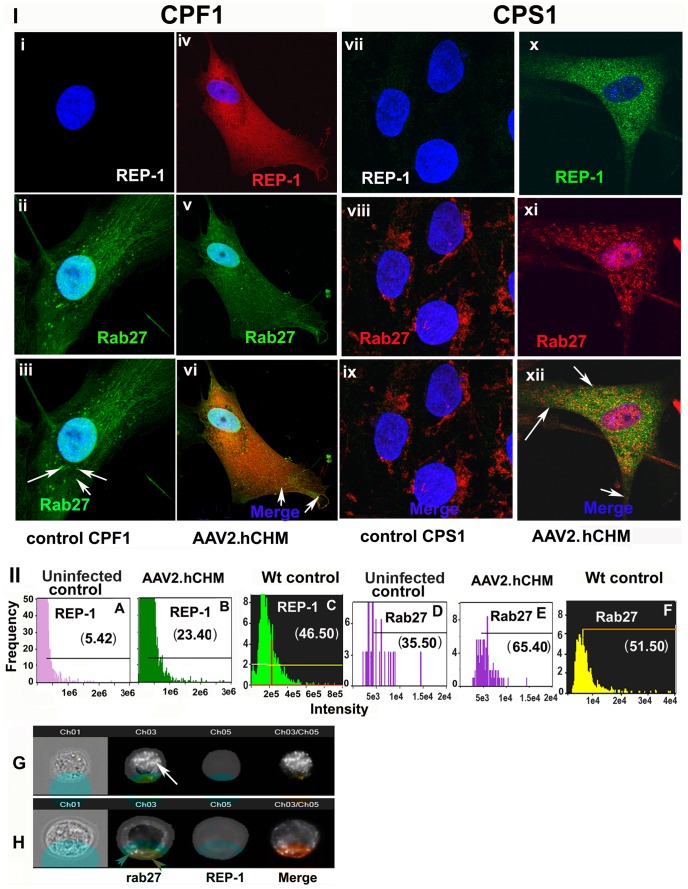
Trafficking of RAB27 protein is restored in affected cells after infection with AAV2. **hCHM.** CPF1 fibroblasts (i–vi) or CPS1 iPSCs (vii–xii) derived from CHM individuals showed improved trafficking of RAB27 after infection with AAV2. hCHM. In control CPF1 (i–iii) or CPS1 (vii–ix) untreated cells, Rab 27a (Green) was localized near the nucleus, whereas infection with AAV2. hCHM favored trafficking of RAB27 out of the perinuclear region in both CPF1(Rep-1 red, RAB27-green) (v–vi) and CPS1 (xi–xii) cells (REP1-green; RAB27-red). Nuclei are stained with DAPI and appear blue. **II).** Quantitative analysis of REP-1 and RAB27 levels in CHM iPSCs measured with imageStream. Histograms represent the increased level of exogenous REP-1 in cells infected with AAV2. hCHM (B) compared to controls (A) and unaffected wt controls (C). However, the level of REP-1 in transduced cells is reduced compared to unaffected control cells (E). Labeled Rab was increased in the surface mask in transduced cells (E) compared to uninfected cells (D). The levels of membrane-associated Rab 27 are comparable to the levels observed in unaffected wild type controls (F) Panel G and H shows representative cell images demonstrating the trafficking of Rab 27 to cell membrane in grey-scale. From left to right are shown: Brightfield, Rab 27, and REP-1, followed by composite images of REP-1 and RAB27. The cell surface masks used to define the inside and surface of the cell are overlayed in Brightfield and RAB/REP-1 labeled cells. White arrow in panel G, accumulated Rab inside the cell. Red arrowheads in panel H, presence of membrane Rab.

To determine whether infection of CHM cells with AAV2. hCHM corrects the primary protein trafficking defect which results from loss of REP-1 function, fibroblasts and iPSC lines were infected with AAV2. hCHM and assessed for changes in trafficking of RAB27, the major target of REP-1. Immunofluorescence analysis of control (untreated, affected) CPF1 and cells where REP-1 protein is absent (figure 5-I-i-iii,vii-ix), demonstrated the localization of RAB27 protein near the nuclear region (figure 5-I-ii-iii;viii-ix) whereas in presence of exogenous REP-1 (figure 5-I-iv-vi;x-xii), RAB27 was found to be trafficked to the membrane (figure 5-I-v-vi;xi-xii).

To further evaluate the details of RAB27 protein distribution in transduced versus untransduced cells, we imaged cells using an Amnis Imagestream II (Amins Corp, Seattle, WA), a flow cytometer equipped with fluorescence microscopy (figure 5-II). The Imagestream collects fluorescent images of every acquired cell and can assess co-localization of fluorescently labeled proteins and subcellular compartments in a quantitative manner. Quantitative analysis of RAB27 trafficking in presence of REP-1 was confirmed by the ImageStream by defining a cell surface mask on the bright field image (figure 5-II). Based on the signal in that particular channel, masks are calculated for each cell. Masking makes it possible to localize a positive protein signal within the cell compared to regular Flow cytometry [40], [41]. In untreated iPSCs, minimal expression of REP-1 was observed (figure 5-II A). In contrast, cells transduced with AAV2. hCHM showed an approximately 4-fold increase in REP-1 levels (figure 5-II B; p = 0.01). Though there is a significant increase in the expression of REP-1, the levels are still lower than the endogenous REP-1 observed in unaffected control cells (figure 5-II-C). With the increase in levels of REP-1, there was also an approximately 2 fold increase compared to baseline in the levels of surface or membrane-associated RAB27 in AAV2. hCHM-treated vs. untreated cells (figure 5-II D,E p = 0.01). Approximately 65% of RAB27 protein was observed to be localized to membranes in AAV-transduced cells (figure 5-II E), while only 35% of Rab protein was on the membrane of untreated cells (figure 5-II D). The membrane RAB27 levels in transduced cells are comparable to the levels of RAB27 in unaffected wild type control cells (figure 5-II F), where 51% of cells expressed RAB27 on the cell membrane. When a mask was used to define the membrane versus interior of the cell, the vast majority of RAB27 protein was localized in the interior in uninfected CHM control cells (figure 5-III G). In comparison, RAB27 protein was on the membrane of AAV2. hCHM transduced CHM cells (figure 5-III H). Thus, these results demonstrate that AAV2-mediated delivery of REP-1 to iPSCs is able to restore trafficking of RAB27 protein to the membrane of the cell. Notably, the high transduction efficiency of AAV2 in iPSCs allows use of the Amnis technology, whereas this approach is not possible when fibroblasts are used as target cells as these are less efficiently transduced by AAV.

### Safety of Infection with AAV2.hCHM *in vitro* and *in vivo*

**Figure 6:**
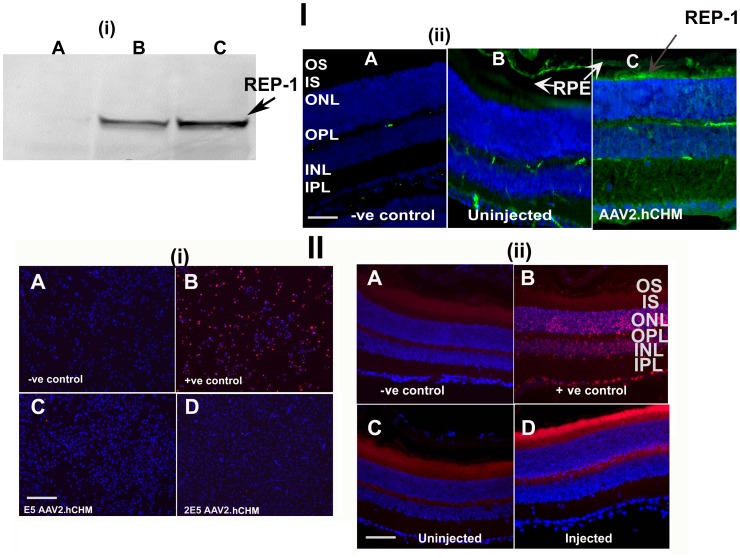
Infection of wild type mouse retinas with AAV2. **hCHM results in transduction of retinal cells and appears safe. I**). Western blot analysis of retinas of normal sighted control mice injected subretinally with 3E6 vg AAV2. hCHM shows one single expected size band in different animals (B, C) (i). A. Uninjected control. Immunolabeling of the AAV2. hCHM-injected retinas (I-ii) shows the localization of the REP-1 protein (Green) to photoreceptors and retinal pigment epithelium (I-ii-C). Nuclei are stained with DAPI. A- negative control, B-uninjected control retina. RPE: Retinal pigment epithelium; OS: Outer segments; IS: Inner segments; ONL: Outer nuclear layer; OPL: Outer plexiform layer; INL: Inner nuclear layer; IPL: Inner plexiform layer. **II).** TUNEL staining of CHO cells (II-i) and retinal sections (II-ii) from retinas injected with AAV2. hCHM in comparison to control uninjected tissue (II)**.** A: CHO cells were infected with E5 (II-i-C), and 2E5 (II-i-D) vg/ml of AAV2. hCHM to evaluate the cytotoxicity of the virus. There were few apoptotic nuclei 72 h after transduction, showing that infection did not result in acute cell death. There was no increase in TUNEL-positivity in AAV2. hCHM injected retinas (II-ii-D). Uninjected (II-ii-C) and injected retina appear similar (II-ii-D).Autofluorescence (red color) is observed in photoreceptor outer segments in all panels. Nuclei are stained with DAPI. Negative controls were generated by incubating the tissue with TUNEL reagents alone (i–A; ii–A). DNAse I treated cells or retinal sections were used as positive controls (i–B; ii–B).

To evaluate the safety of *in vivo* expression of AAV2. hCHM, wild type (C57Bl/6) mice were injected subretinally with the rAAV vector. Three weeks post injection, retinal tissues were collected for protein analysis and histology. Immunoblot of the retinal tissue with the human REP-1 specific, 2F1 antibody confirmed the presence of REP-1 protein of the expected size (figure 6-I-i). Immunofluorescence analyses of the retinal sections with 2F1 antibody further confirmed the expression/localization of REP-1 protein to the inner segment and outer nuclear layers of photoreceptors (figure 6-I-iiC).

To screen for short-term toxicity resulting from infection with AAV2. hCHM, CHO cells were evaluated for apoptosis by TUNEL staining following infection at MOI 1×10^5^ or 2×10^5^ of AAV2. hCHM. Neither dose of AAV2. hCHM resulted in cell death (figure 6-II-i). Both samples exposed to AAV2. hCHM and those that were studied as untreated controls showed similar viability (>97% viable) (figure 6-II-i). There were also similar (low) numbers of apoptotic cells (2–3%) in AAV-treated (and uninfected) cells compared to positive controls (figure 6-II-i).

The retinae of animals injected with AAV2. hCHM showed similar (low) levels of apoptotic cell death as control (untreated) mouse retinae (figure 6-II-iiD vs C). The number of rows of nuclei in the outer nuclear layer was similar in treated and control mice indicating that there were no short-term degenerative changes in photoreceptors resulting from over-expression of AAV2. hCHM (figure 6-II-ii). There was no evidence of inflammatory infiltrate in these tissues as judged by hematoxylin and eosin staining (figure B in file S1).

In summary, the results of subretinal delivery of AAV2. hCHM show that transfer of the REP-1-encoding gene to retinal cells results in high levels of expression without significant evidence of toxicity.

Any additional data relevant to these studies that has not been submitted for publication can be obtained by request to the authors.

## Discussion

Choroideremia has several attributes of an optimal target for gene augmentation therapy: The cDNA has been cloned and can be packaged into rAAV, a vector with extensive laboratory and clinical safety and efficacy data in human clinical trials for retinal degenerative disease. The disease is slowly but inexorably progressive, with a defined window of opportunity for intervention. The pathogenetic mechanisms responsible for the disease have been described and the pathology is limited to the retina. As described above, the disease is symmetrical from eye to eye, allowing design of studies which optimize the risk:benefit ratio. Further, the clinical features and course of the disease have been well characterized, allowing one to design appropriate outcome measures for a clinical trial.

The main challenge at present to developing proof-of-concept data for moving forward to a clinical trial is the lack of an animal model which has similar functional and morphologic features as the human retina. Knockout of the murine *Chm* gene is embryonic lethal. A conditional knock-out approach was tested using the Cre/loxP system of site-specific recombination and a transgenic line expressing tamoxifen-regulated MerCreMer. This led to 3 Rep1 alleles inherited by female offspring of tamoxifen-treated Rep13loxP/Y, Cre+ males. The heterozygous null female carriers (*Rep1* null/WT and *Rep1*null+*Neo*/WT) exhibit many of the features of CHM [42]. Additional animals were generated using a tissue-specific Cre expression in order to show the differential effects of *Chm* knockout in RPE vs. photoreceptors [43]. While the models were successful, the degeneration in all of the different lines progresses slowly. Further, availability of viable animals is limited in large part due to the complicated method of their generation, making them difficult “subjects” to obtain for proof-of-concept studies. They have been used by one group in proof-of-concept studies using recombinant lentivirus [44].

Because of challenges of the animal model, we proceeded to explore *in vitro* approaches to obtaining proof-of-concept of AAV2-mediated gene augmentation therapy for CHM. There are various limitations imposed by the cell lines that have already been developed [30]. Lymphoblasts are difficult to transduce using AAV2 (figure C in file S1). Primary fibroblasts can be transduced by AAV2 although this is an inefficient technique and, they grow slowly and only for a limited number of generations. Due to the invasiveness of the biopsy necessary to obtain the fibroblasts, their availability and numbers are limited. Reprogramming of somatic cells (obtained through phlebotomy) represents a novel approach to obtaining patient-specific stem cells harboring individual disease mutations. Because of the unlimited replicative capacity and clonability, iPSCs can provide adequate material for understanding the disease pathology and developing sustained treatment approaches. We therefore explored the possibility of developing iPSCs as a preclinical model. The advantages of iPSCs include the fact that they can be developed from a wide variety of tissue sources, including white blood cells obtained through phlebotomy or from cells that had already been banked, thus minimizing repeated invasive procedures. These cells do not undergo senescence and they have an unlimited lifespan. Further, they can be differentiated in a tissue-specific fashion to generate *in vitro* tissue models [45], [46]. Before our study, it was not known whether iPSCs could be transduced using AAV2. Here we show that not only can they be efficiently transduced with AAV2, but that transduction of CHM cells with a wild type *hCHM* cDNA results in functional restoration of REP1-mediated enzymatic activity and protein trafficking. Transduction was far more efficient in iPSCs compared to fibroblasts. Further, because we could generate far more iPSCs than fibroblasts, we could use iPSCs to characterize the biochemical and cellular responses in detail. Finally, since iPSCs can be generated easily from any patient, one can use these to compare and evaluate different variables which may affect treatment efficacy, such as the nature of the disease-causing mutation.

Using a “personalized medicine” approach, we studied cells from one patient in whom a premature stop codon prevented formation of the intact REP-1 protein and cells from another patient who had a *CHM* missense mutation. The iPSC rescues in both of these samples showed conclusively that both of these *CHM* mutations cause disease through a lack-of-function mechanism. REP-1 protein is involved in the prenylation of Rab proteins which, in turn are essential for phagocytosis, and intracellular trafficking [39], [47]. RAB27 is a protein found in high levels in the RPE and choriocapillaris and accumulates in the cytosol (instead of its normal localization to the membrane) in cells of CHM patients [16]. In the studies reported here, we show that production of wildtype REP-1 protein after infection with AAV2. hCHM allows affected cells to prenylate RAB27 which then corrects the trafficking defect associated with loss of REP-1 function.

We had previously used transformed lymphoblast and primary fibroblast cells from the CPF1 (premature stop codon) family to demonstrate rescue of the CHM phenotype using a first generation recombinant adenovirus, [30] a virus which results in high levels and rapid onset of transgene expression. Gene transfer in that situation was effective but the adenoviral vector was not moved to clinical trial because it does not result in stable expression [48] and also can elicit a toxic inflammatory response [49]. In the present study, we generated a vector using components very similar to those of the vector that we used successfully in Phase I/II studies in a clinical trial for Leber Congenital Amaurosis due to *RPE65* mutations (LCA-RPE65), an AAV vector with serotype 2 capsid [25], [50], [51], [52]. This vector is also the first to be used in a Phase III study for LCA-RPE65 (clinicaltrials.gov #NCT00999609).

Recombinant AAV has an excellent safety profile. It has been or will shortly be used in more than 191 subjects in ocular gene therapy clinical trials (clinicaltrials.gov). Wild-type AAV has never been shown to cause human (or animal) disease. Immune response to rAAV is favorable since rAAV lacks any virus-derived open reading frames. The only AAV proteins which come in contact with the target tissue are those in the capsid. Those proteins do not elicit a cytotoxic T cell response in animals or humans even under prime-boost conditions (readministration) [24], [25]. These data reveal that infection of primary cell lines, iPSCs and retinal cells *in vivo* with AAV2. hCHM resulted in high levels of wild-type REP-1 protein and that the transduction reversed the defects in trafficking and prenylation of Rab proteins in the affected cells. Future studies could unravel details of the pathogenetic mechanism of choroideremia by testing (and then rescuing) iPSCs that have been differentiated into RPE cells.

In summary, we have generated a rAAV vector capable of introducing a functional version of the human *REP-1* gene *in vitro* in cells from affected patients. Transduction of human REP-1-deficient cells with this vector provided stable expression of functional REP-1 that not only improves the trafficking of accumulated Rab 27a proteins but also prenylates the proteins. Expression of REP-1 did not produce cytotoxicity in cells *in vitro* or *in vivo*. These data provide the platform for moving forward to develop a human clinical trial for choroideremia.

## Supporting Information

File S1 (TIF)
