# Correction: AAV-Mediated Gene Therapy for Choroideremia: Preclinical Studies in Personalized Models

**DOI:** 10.1371/journal.pone.0129982

**Published:** 2015-06-19

**Authors:** Vidyullatha Vasireddy, Jason A. Mills, Rajashekhar Gaddameedi, Etiena Basner-Tschakarjan, Monika Kohnke, Aaron D. Black, Krill Alexandrov, Shangzhen Zhou, Albert M. Maguire, Daniel C. Chung, Helen Mac, Lisa Sullivan, Paul Gadue, Jeannette L. Bennicelli, Deborah L. French, Jean Bennett

In “AAV-mediated gene therapy for Choroideremia: Preclinical studies in personalized in vitro models”, by Vasireddy et al (DOI: 10.1371/journal.pone.0061396), two panels in Fig 1 contained immunoblot images that were spliced together by the authors for the sake of re-ordering lanes and omitting unnecessary data on the published figure. The authors apologize for not acknowledging these manipulations in figure preparation in the original Figure legend.

In the corrected version of Fig 1, we present the same data as those published in the original article. However, panels (IIi) and (III) have been modified so that black, vertical lines clearly indicate where lanes have been spliced together during figure preparation. To demonstrate the validity of the data in the affected panels, we present the original gel images in Supporting Figs 1 and 2. The figure legends clearly indicate which lanes from the original gels are included in the published figure. See the original article for detailed Methods and a discussion of the Results. We have also replicated the Fig 1 immunoblot experiments in full (including the transfections and lysate preparations) but placed the samples in the correct order in the new gels. Those unmanipulated blot images are presented as Fig 2 of this Correction.

**Fig 1:**
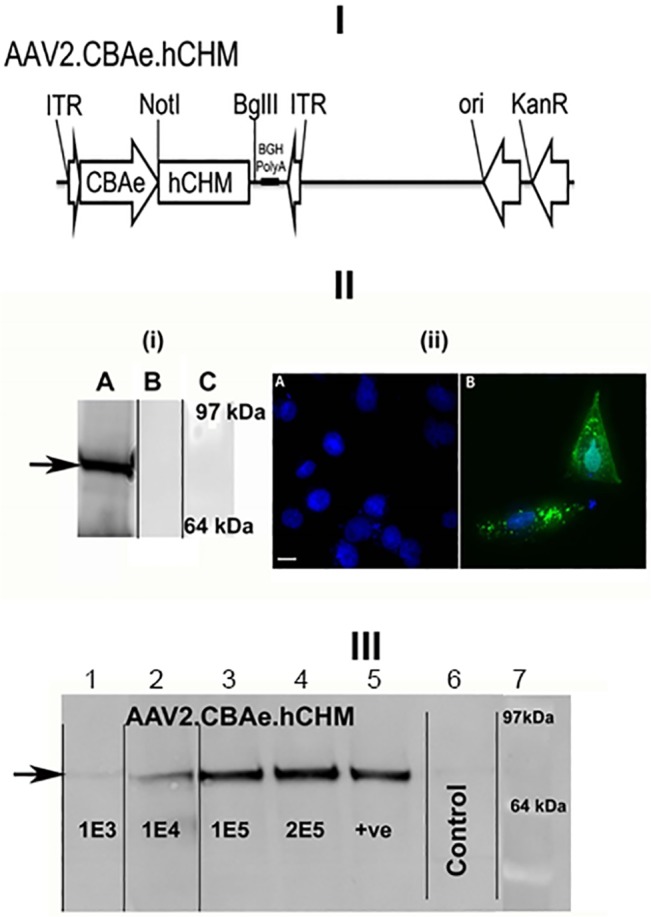
Generation and Characterization of AAV2. hCHM. I). Schematic of the AAV proviral plasmid carrying human *CHM*. under the control of the cytomegalovirus enhancer chicken beta actin (eCBA) promoter. ITR: Inverted terminal repeats; Ori: Replication origin; KanR: Kanamycin resistance gene. II) i) Immunoblot and ii) fluorescent analysis reveals REP-1 protein in CHO cells transfected with pAAV2.hCHM. Lane A: Transfected cell (25 μg protein), B: Control (untransfected) cells, C- protein marker. Immunocytochemical analysis revealed the localization of REP-1 to the cytosolic region (II-ii-B; Green). No REP1 is observed in control cells (II-ii-A). Nuclei are stained with DAPI and appear blue. Scale bar is 50 μM. III) Immunoblot analysis of CHO cells infected with 1E3-2E5 viral genomes (vg) of AAV2. hCHM shows an increase in REP-1 protein (indicated by arrow) proportional to the titer. Positive (+ve) control: pAAV2. hCHM-transfected CHO cell lysate. Black lines in panels IIi and III indicate where lanes from original immunoblots have been spliced together during figure preparation *(original legend is reproduced from the published PLOS ONE article)*.

**Fig 2:**
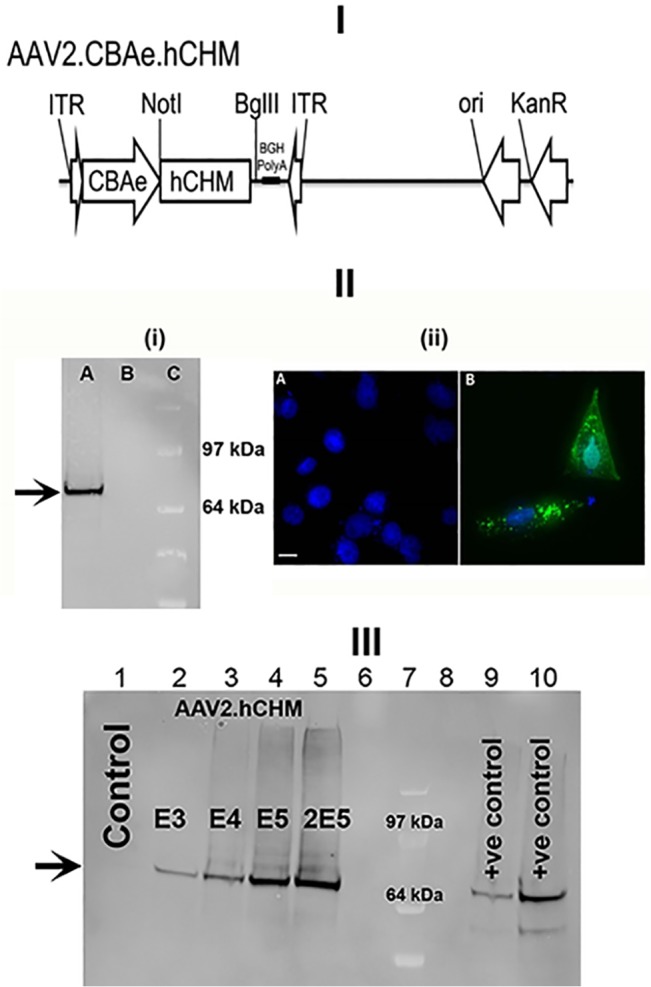
Revised Fig 1, with additional immunoblots made using a complete replication of the experiment described in the original Fig 1 (including the transfections and lysate preparations) but with samples loaded in the correct consecutive order on the gel. I). Schematic of the AAV proviral plasmid carrying human *CHM* under the control of the cytomegalovirus enhancer chicken beta actin (eCBA) promoter. ITR: Inverted terminal repeats; Ori: Replication origin; KanR: Kanamycin resistance gene. II) i) Immunoblot and ii) fluorescent analysis reveals REP-1 protein in CHO cells transfected with pAAV2.hCHM. Lane A: Transfected cell (25 μg protein), B: Control (untransfected) cells, C- protein marker (SeeBlue Plus2, Invitrogen, Grand Island, NY). Immunocytochemical analysis revealed the localization of REP-1 to the cytosolic region (II-ii-B; Green). No REP1 is observed in control cells (II-ii-A). Nuclei are stained with DAPI and appear blue. Scale bar is 50 μM. III) Immunoblot analysis of CHO cells infected with 1E3-2E5 viral genomes (vg) of AAV2. hCHM (lanes 2–5) show an increase in REP-1 protein (indicated by arrow) proportional to the titer. Lane 1 is a negative control containing lysate from uninfected CHO cells. Positive (+ve) controls: pAAV2. hCHM-transfected CHO cell lysates (lanes 9, 10). Lanes 6 and 8 were not loaded. Lane 7 contains the SeeBlue Plus 2 protein marker.

## Supporting Information

S1 Fig Raw, unaltered immunoblot image (using the human REP-1-specific antibody, antibody, 2F1) used to make Fig 1IIi. Lane 12 contains a protein marker (SeeBlue Plus2, Invitrogen, Grand Island, NY), lanes 2–6 and 9–10 show results of loading 25 μg of CHO cell lysate after replicate transfections with pAAV2.hCHM, and lane 1 was blank. Lanes 7, 8 and 11 show untreated control CHO cell lysates. Lysates from cells that were floating after transfection are shown in lanes 3 and 6. **Lanes 4, 7, and 12 were presented in Fig 1IIi lanes A, B, and C, respectively.
(TIF)

S2 Fig Raw, unaltered immunoblot image (using the human REP-1-specific antibody, antibody, 2F1) used to make Fig 1III. Lanes 1, 3, 4, and 6 contained lysates of CHO cells infected with 1E4, 1E5, 2E5 and 1E3 vg of AAV2.hCHM, respectively. Lane 5 is a positive control (pAAV2.hCHM-transfected CHO cell lysate). Lane 2 was not used and lane 7 was lysate from untreated CHO cells. Lanes 8 and 9 contained samples from an unrelated experiment and lane 10 contained the SeeBlue Plus2 protein marker. **Lanes 6, 1, 3, 4, 5, 7, 10 were presented in Fig 1III lanes 1, 2, 3, 4, 5, 6, 7, respectively in order to present the immunoblot results according to increase in AAV2.hCHM titer. The irrelevant lanes (original lanes 2, 8, and 9) were not shown in the original Fig 1III.
(TIF)
