# Supplementary figures and images for: AAV-Mediated Gene Therapy for Choroideremia: Preclinical Studies in Personalized Models
Source: PLoS One. 2013 May 7;8(5):e61396. doi: 10.1371/journal.pone.0061396 (PMC3646845; doi:10.1371/journal.pone.0061396)

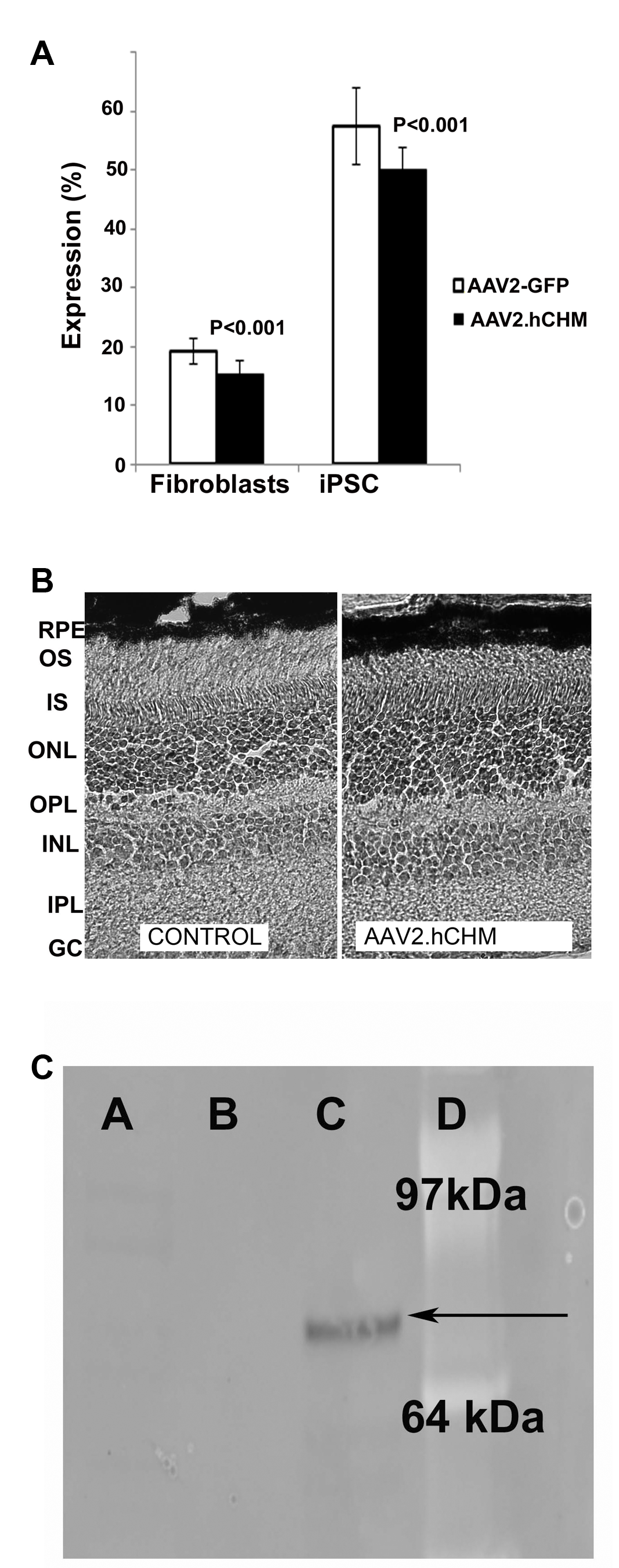

Supplement: File S1 — (TIF) [file pone.0061396.s001.tif]
