# Supplementary figures and images for: Correction: AAV-Mediated Gene Therapy for Choroideremia: Preclinical Studies in Personalized Models
Source: PLoS One. 2015 Jun 19;10(6):e0129982. doi: 10.1371/journal.pone.0129982 (PMC4474960; doi:10.1371/journal.pone.0129982)

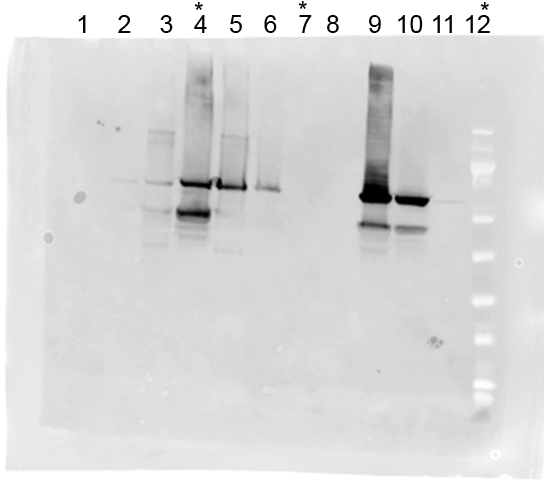

Supplement: S1 Fig — Lane 12 contains a protein marker (SeeBlue Plus2, Invitrogen, Grand Island, NY), lanes 2–6 and 9–10 show results of loading 25 μg of CHO cell lysate after replicate transfections with pAAV2.hCHM, and lane 1 was blank. Lanes 7, 8 and 11 show untreated control CHO cell lysates. Lysates from cells that were floating after transfection are shown in lanes 3 and 6. **Lanes 4, 7, and 12 were presented in Fig 1IIi lanes A, B, and C, respectively. (TIF) [file pone.0129982.s001.tif]

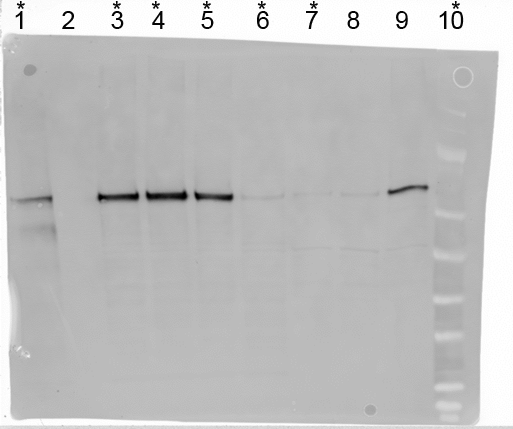

Supplement: S2 Fig — Lanes 1, 3, 4, and 6 contained lysates of CHO cells infected with 1E4, 1E5, 2E5 and 1E3 vg of AAV2.hCHM, respectively. Lane 5 is a positive control (pAAV2.hCHM-transfected CHO cell lysate). Lane 2 was not used and lane 7 was lysate from untreated CHO cells. Lanes 8 and 9 contained samples from an unrelated experiment and lane 10 contained the SeeBlue Plus2 protein marker. **Lanes 6, 1, 3, 4, 5, 7, 10 were presented in Fig 1III lanes 1, 2, 3, 4, 5, 6, 7, respectively in order to present the immunoblot results according to increase in AAV2.hCHM titer. The irrelevant lanes (original lanes 2, 8, and 9) were not shown in the original Fig 1III. (TIF) [file pone.0129982.s002.tif]
